# Microperimetry and multimodal imaging in polypoidal choroidal vasculopathy

**DOI:** 10.1038/s41598-018-33781-5

**Published:** 2018-10-25

**Authors:** Jennifer H. Acton, Ken Ogino, Yumiko Akagi, John M. Wild, Nagahisa Yoshimura

**Affiliations:** 1 College of Biomedical and Life Sciences, Cardiff University, Maindy Road, Cardiff, Wales CF24 4HQ UK; 2 Department of Ophthalmology and Visual Sciences, Kyoto University Graduate School of Medicine, Kyoto, Japan

## Abstract

Polypoidal choroidal vasculopathy (PCV) is a degenerative macular disease. The study determined the topographical concordance in the areal extent of PCV, defined by indocyanine green angiography (ICGA), and the corresponding outcomes from spectral-domain optical coherence tomography (SD-OCT) and microperimetry, in 25 individuals (25 eyes) who had undergone 3 months of anti-vascular endothelial growth factor treatment. The differential light sensitivity within 10° eccentricity was evaluated by Pattern Deviation probability analysis. The concordances and proportional areal extents of the abnormality for ICGA, SD-OCT and microperimetry were compared. The concordance in the areal extent between all three modalities was 59%. The median concordance between ICGA and microperimetry was 60%; between ICGA and SD-OCT, 70%; and between SD-OCT and microperimetry, 72%. SD-OCT and microperimetry each identified a greater areal extent (>20%) compared to ICGA in 13 and 19 eyes, respectively. A greater areal extent (>20%) was present in 9 eyes for microperimetry compared to SD-OCT and in 5 eyes for SD-OCT compared to microperimetry. SD-OCT and microperimetry each identified a greater area of abnormality than ICGA which supports the clinical utility of SD-OCT. Strong concordance was present between SD-OCT and microperimetry; however, microperimetry identified additional areas of functional abnormality.

## Introduction

Polypoidal choroidal vasculopathy (PCV) is a progressive disease of the choroidal vasculature^1^. The diagnosis is based upon the presence of hyperfluorescent sub-retinal polypoidal lesions, with or without a branching vascular network^2,3^, visible by indocyanine green angiography (ICGA). Associated clinical features include subretinal haemorrhage; serosanguinous detachment of the retinal pigment epithelium at the macula and/ or peripapillary region; and subretinal fluid^4,5^.

PCV is considered to be either a distinct clinical entity^1,4^ or a subtype of Type 1 choroidal neovascularization^6^. A further distinction can be made between idiopathic PCV and secondary polyps associated with neovascular age-related macular degeneration (AMD)^7^. The estimated prevalence of PCV in neovascular AMD is 8–10% in Caucasians^5^ and 41% in Japanese individuals^8^.

The choroid, by spectral-domain optical coherence tomography (SD-OCT), is typically thicker than normal in PCV^9^. SD-OCT^10,11^ and swept-source OCT angiography^12^ are both effective in detecting the polypoidal lesion, but are not advocated as a replacement for ICGA in the differentiation of PCV from AMD^12^.

Most clinical trials involving the efficacy of photodynamic therapy and/or anti-vascular endothelial growth factor (VEGF) therapy in the treatment of PCV have evaluated functional recovery solely in terms of visual acuity and have not considered para- and/or peri-foveal function^13–15^. Several studies have used the outcomes from either standard automated perimetry^16^ or microperimetry^17–19^ or from focal electroretinography of the macula^17,20,21^; nevertheless, the clinical impact of these functional assessments remain equivocal. Microperimetry incorporates two novel features, compared to standard automated perimetry, which makes it particularly suitable for the assessment of macular disease. It determines the differential light sensitivity whilst simultaneously providing an infra-red fundus image and uses eye tracking to correct for unsteady and/or non-central fixation.

Although the structural appearance of PCV by ICGA is well documented^10,11^, the topographical concordances in the extent of the lesion by ICGA, SD-OCT and microperimetry are unknown and the potential clinical impact of microperimetry remains unclear^22^. The aim of the study, therefore, was to determine the topographical concordance in the areal extents of the abnormality in PCV, defined by ICGA, SD-OCT and microperimetry, in individuals who had undergone 3 months of anti-VEGF therapy.

## Results

The median age was 73 years (IQR 69, 77; range 63–88 years). Twenty-three eyes were undergoing ranibizumab therapy and 2 eyes bevacizumab therapy.

### PCV characteristics

**Figure 1:**
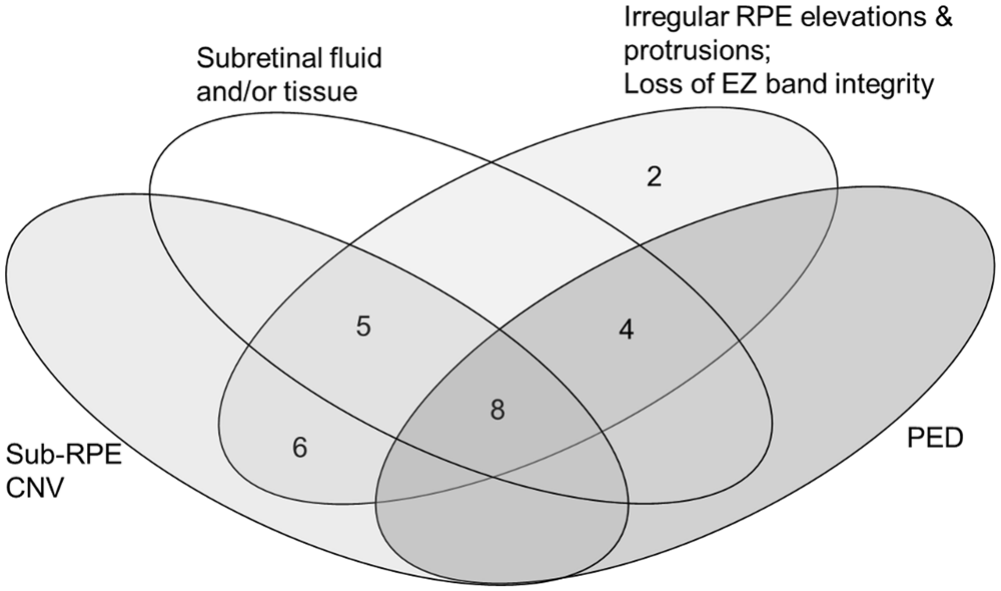
The frequency of, and the relationship between, the associated macular abnormalities for the 25 eyes with polypoidal choroidal vasculopathy. RPE = retinal pigment epithelium; CNV = choroidal neovascularization; EZ = ellipsoid zone; PED = pigment epithelial detachment.

All 25 eyes exhibited disruption to the inner segment ellipsoid zone; 19 eyes exhibited sub-retinal pigment epithelium choroidal neovascularization; 17 subretinal fluid and/or subretinal tissue; and 12 pigment epithelial detachment (Fig. 1).

Sixteen eyes had exudative changes associated with the branching vascular network lesion. The median areal extents of the abnormality by Total Deviation (TD) and by SD-OCT were significantly larger in these eyes compared to the 9 eyes without such changes (Mann-Whitney, p = 0.023 and p = 0.008, respectively) (Table 1).

**Table 1:** Microperimetry, SD-OCT and ICGA outcomes.

	Eyes with exudative changes associated with branching vascular network lesion n* = *16	Eyes without exudative changes associated with branching vascular network lesion n* = *9	All eyes n* = *25
**Visual Acuity (logMAR)** Median (IQR)	0.3 (0.1, 0.6)	0.1 (0.1, 0.2)	0.2 (0.0, 0.3)
**Total Deviation (dB)** Median (IQR)	−10.3 (−15.1, −6.7)	−5.6 (−8.0, −5.0)	−8.3 (−19.1, −4.6)
**Mean PD** **(dB)** Median (IQR)	−4.8 (−6.5, 3.0)	−4.5 (−5.8, −3.1)	−4.6 (−9.2, −3.0)
**Mean number of PD defects ≤1% (locations)** Median (IQR)	38 (35, 46)	34 (30, 37)	38 (21, 42)
**Total retinal thickness (μm)** Median (IQR)	302 (275, 307)	279 (266, 289)	287 (248, 306)
**Choroidal thickness (μm)** Median (IQR)	250 (188, 316)	280 (150, 320)	280 (120, 320)
**SD-OCT lesion area (mm**^2^**)** Median (IQR)	13.9 (9.2, 16.5)	6.9 (6.0, 9.1)	10.2 (2.6, 14.5)
**ICGA lesion area (mm**^2^**)** Median (IQR)	12.2 (6.4, 17.0)	9.1 (3.0, 10.9)	10.0 (4.3, 14.0)

IQR = interquartile range; ICGA = indocyanine green angiography; SD-OCT = spectral domain optical coherence tomography; PD = Pattern Deviation.

**Figure 2:**
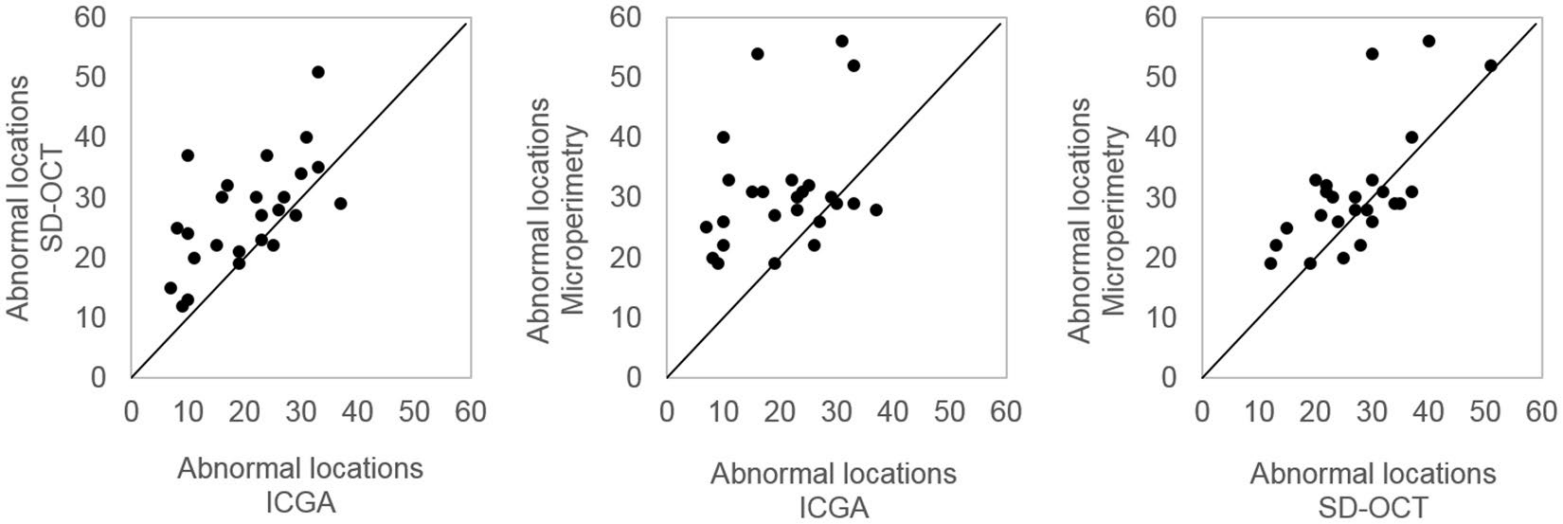
The number of abnormal locations, for each pair of modalities, for the 25 eyes with polypoidal choroidal vasculopathy. The solid line represents the line of unity. SD-OCT = spectral-domain optical coherence tomography; ICGA = indocyanine green angiography.

Twenty eyes had a greater areal extent by SD-OCT (Fig. 2) compared to ICGA, 19 a greater areal extent by microperimetry compared to ICGA and 16 by microperimetry compared to SD-OCT. Stimulus locations exhibiting an abnormal sensitivity were contiguous. The percentage of individuals with abnormal sensitivity of p ≤ 5% ranged from 76–100% at locations within an eccentricity of 4° and ranged from 44–92%, between 5° and 10°.

### Concordances and areal extents between modalities

**Figure 3:**
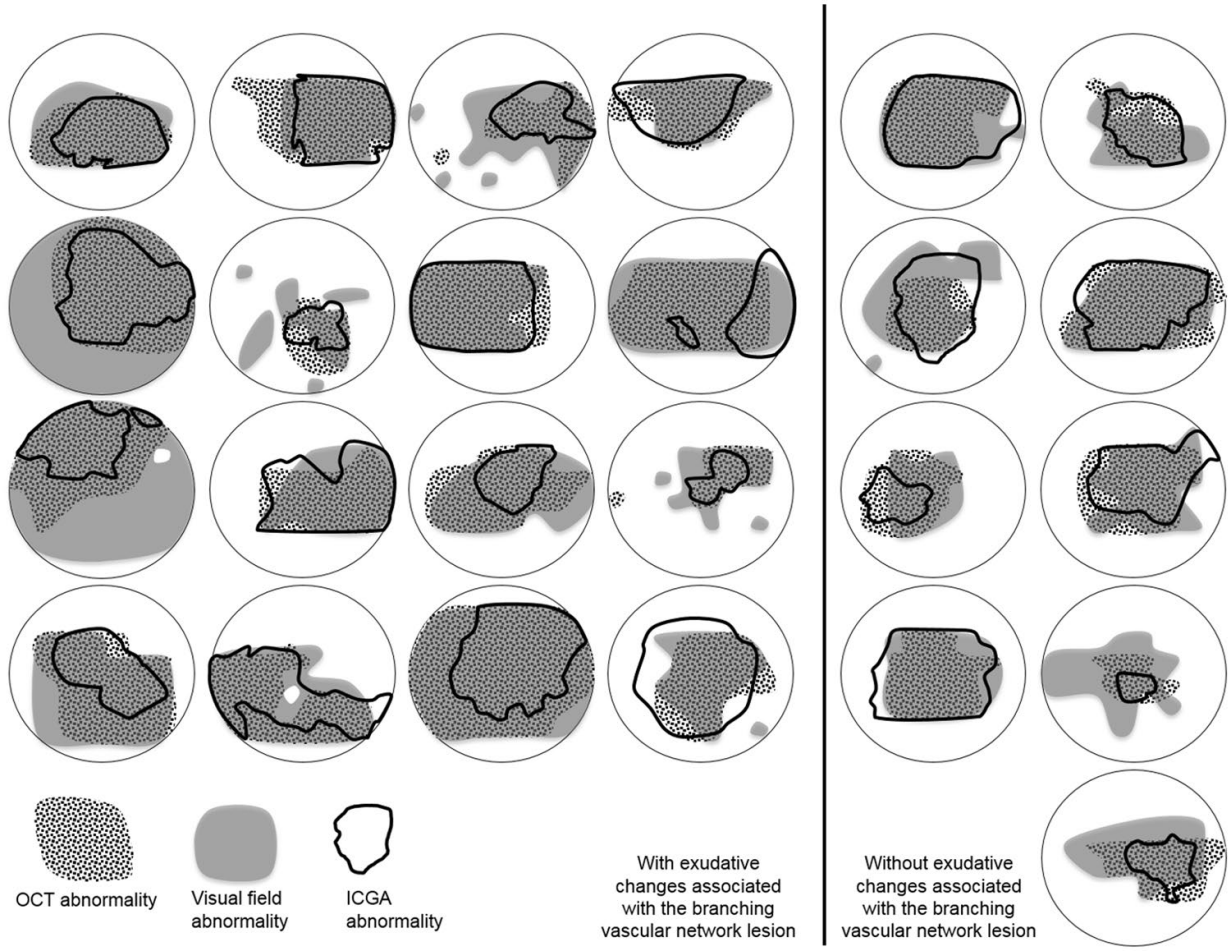
A schematic representation of the overlay of the areal extent of the abnormality by three modalities. The schematic is scaled within 10° eccentricity, and depicts the abnormality by microperimetry (solid grey shading), by SD-OCT (dotted shading), and by ICGA (within the black line boundary). The 16 eyes with exudative changes associated with the branching vascular network lesion (left) and the 9 eyes without these exudative changes (right) are shown. SD-OCT = spectral- domain optical coherence tomography; PCV = polypoidal choroidal vasculopathy; ICGA = indocyanine green angiography.

The median concordance for the areal extent of the abnormality by all three modalities was 59% both for the 16 eyes with exudative changes associated with the branching vascular network and also for the 9 eyes without these changes. The summary measures for the various concordances are given in Table 2 and are illustrated for each individual in Fig. 3. Eight eyes displayed a concordance of <35% between the three modalities: of these, 5 eyes exhibited abnormality by microperimetry which was not evident by either SD-OCT or ICGA.

**Table 2:** Concordances in the areal extent of the lesion by microperimetry, SD-OCT and ICGA (See Supplementary File 1).

	Eyes with exudative changes associated with branching vascular network lesion n = 16	Eyes without exudative changes associated with branching vascular network lesion n = 9	All eyes n = 25
**MP, SD-OCT, ICGA**			
**Concordance (%):** Median (IQR)	59 (33, 65)	59 (29, 74)	59 (17, 66)
**MP, ICGA**			
**Concordance (%):** Median (IQR)	59 (44, 71)	67 (35, 77)	60 (19, 71)
Greater areal extent by MP (eyes)	13	6	19
Greater areal extent by ICGA (eyes)	2	1	3
**SD-OCT, ICGA**			
**Concordance (%):** Median (IQR)	68 (53, 80)	81 (47, 90)	70 (21, 85)
Greater areal extent by SD-OCT (eyes)	9	4	13
Greater areal extent by ICGA (eyes)	1	0	1
**MP, SD-OCT**			
**Concordance (%):** Median (IQR)	74 (64, 83)	67 (61, 86)	72 (35, 84)
Greater areal extent by MP (eyes)	6	3	9
Greater areal extent by SD-OCT (eyes)	3	2	5

MP = microperimetry; ICGA = indocyanine green angiography; SD-OCT = spectral domain optical coherence tomography; IQR = interquartile range.

#### ICGA and OCT

The median concordance for the 25 eyes in the areal extent of the lesion between ICGA and SD-OCT was 70%. The SD-OCT area of abnormality extended beyond that by ICGA by a median of 23.1% (IQR 13.6, 46.7). The additional area exceeded 20% in 13 eyes. Conversely, in one eye the ICGA lesion was greater by at least 20% (see Supplementary File 1).

#### ICGA and Microperimetry

The median concordance in the areal extent of the lesion by ICGA and the visual field loss was 60%. The visual field loss extended beyond the ICGA area of abnormality by a median of 33.3% IQR (22.2, 60.0). The additional area exceeded 20% in 19 eyes. In one of these 19 eyes, the ICGA lesion also extended beyond the visual field loss by at least 20%. Conversely, in 3 eyes the ICGA lesion was greater by at least 20%. Microperimetry thus identified a greater areal loss in approximately six times as many eyes compared to ICGA (i.e. 19 compared to 3 eyes).

#### OCT and Microperimetry

The median concordance in the areal extent of the lesion by SD-OCT and the visual field loss was 72%. The visual field loss extended beyond the SD-OCT area of abnormality by a median of 10% (IQR 6.5, 28.6). The additional area exceeded 20% in 9 eyes (median 41%, IQR 29, 43). The SD-OCT area of abnormality also extended beyond the visual field loss by a median of 11.8% (IQR 0.0, 16.1). The additional area exceeded 20% in 5 eyes (median 24%, IQR 23, 27).

#### Retinal and subfoveal choroidal thickness

The sum of the Pattern Deviation (PD) values was negatively correlated with the corresponding sum of the total retinal thicknesses within the area of abnormality (R^2^ = 0.29, p < 0.001). The correlation was higher within the area of concordance (R^2^ = 0.62, p < 0.001). The correlation between the subfoveal choroidal thickness and the mean PD of the locations within 1.4° eccentricity was not statistically significant from zero (R^2^ = 0.15, p = 0.082).

## Discussion

SD-OCT and microperimetry each identified a greater area (>20%) of abnormality than ICGA. Such an outcome suggests that ICGA, alone, may underestimate the size of the lesion in PCV. In addition, microperimetry identified 9 eyes with a greater areal loss relative to SD-OCT, whilst SD-OCT exhibited a greater area of abnormality in 5 eyes. This finding supports the need for both modalities in the evaluation of PCV.

The 70% concordance between SD-OCT and ICGA together with the larger number of eyes exhibiting a greater lesion area by SD-OCT is in agreement with the established clinical utility of SD-OCT in PCV^10,11^. The 72% concordance between microperimetry and SD-OCT is also clinically reassuring. The two concordances, when considered together, validate the concept that microperimetry yields additional clinical information relative to ICGA; in this instance, a sixfold number of eyes exhibiting a greater areal abnormality. Overall, such findings might be expected in that ICGA primarily provides a measure of choroidal vascular integrity; SD-OCT identifies the depth of retinal layer abnormality; and microperimetry, although not identifying the type of lesion, in this context, determines retinal functional integrity. It is likely that a consideration of the field loss in terms of defect depth would further enhance the impact of microperimetry.

The subfoveal choroidal thickness (mean 251 μm, SD 100 μm) lies within the range of that found previously for PCV with the Spectralis SD-OCT in eyes treated with anti-VEGF therapy (mean 326 μm, SD 100 μm^23^) and in treatment naïve eyes (mean 243 μm, SD 93 μm^9^ to 438 μm, SD 88 μm^24^). The limited relationship between choroidal thickness and the mean PD within 1.4° eccentricity is also consistent with that found previously for the mean sensitivity visual field index^18^.

The group mean Mean Sensitivity index for the field loss was similar to those undergoing anti-VEGF therapy in PCV^17,18^.

A strength of the study is that the concordances were evaluated on a location by location basis across the retina and visual field, respectively, rather than simply in terms of a single summary measure of the outcome from each modality. The findings could be influenced by the definition of visual field loss. The study is the first to use PD probability analysis in PCV and is more robust than the use of raw values of sensitivity. PD probability analysis is the gold standard for the delineation of focal visual field abnormality^25^. This technique is age-corrected and also removes any diffuse component of visual field loss.

The focal loss was considered in discrete areas designated around each stimulus location. The region in between stimuli at which potential areas of loss could not be identified corresponded to a maximum diameter of 1.2°.

The study was undertaken at the 3 month loading phase to overcome the known influence of the learning effect associated with microperimetry in individuals with AMD, whereby sensitivity increases from the first to the second examination^26^. However, the findings should also be considered in the context of potential improvement in the morphological and/or functional outcomes arising from the anti-VEGF therapy. The greater loss by microperimetry may have resulted from a slower functional recovery relative to the morphological recovery.

The differences in the areal extents of the lesion between modalities was considered using an arbitrary criterion of 20%. A less conservative criterion of 10% yielded similar outcomes for microperimetry compared to ICGA and for SD-OCT compared to ICGA. However, for microperimetry and SD-OCT, an equivalent number of eyes exhibited greater areal losses with this criterion.

The concordance between each of the three modalities was evaluated on an individual basis in terms of a relative measure which did not require consideration of axial length-dependent image scaling. Axial length influences the superimposition of the stimulus grid onto the infrared fundus image of the MP-1 since the scaling estimation is based solely upon spherical refractive error; however, such variations in the superimposition do not exceed the magnitude of the inter-stimulus separation (1.4°–2.8°). The scaling estimation of the Spectralis instrument is also based on the spherical refractive error, alone, and such discrepancies are similarly negligible.

Although the case series comprised 25 individuals, the diagnosis was robust and unequivocal over a typical range of lesion extents. The evidence base is sufficiently strong to provide proof of concept that differential light sensitivity is of value in the assessment of visual function in PCV.

In conclusion, although ICGA is necessary to identify the features of PCV^2,3,12^, SD-OCT and microperimetry each identified a greater areal extent of abnormality compared to ICGA. The findings confirm the clinical utility of SD-OCT in the management of PCV. Whilst good concordance was present between SD-OCT and microperimetry, the additional areas of abnormality were more pronounced by microperimetry. The latter suggests that functional assessment is a further useful adjunct in the clinical evaluation of PCV.

## Methods

A case series of 25 individuals (18 males) with a diagnosis, in at least one eye, of PCV involving the macular region, only, and who conformed to pre-defined inclusion criteria, was identified from those attending the Department of Ophthalmology and Visual Sciences, Kyoto University Hospital, Kyoto, Japan.

PCV was defined as the presence of one or more hyperfluorescent polypoidal lesions evidenced by ICGA, with or without branching of the vascular network, and with or without subretinal haemorrhage, serosanguineous retinal pigment epithelial detachment, subretinal exudation or serous retinal detachment^27^.

Inclusion criteria comprised high quality images in each eye from fundus photography (TRC-50LX, Topcon, Tokyo, Japan); infrared, ICGA, fluorescein angiography and SD-OCT (Spectralis SD-OCT + cSLO Heidelberg Engineering, Heidelberg, Germany); and a reliable outcome from microperimetry (MP-1 Nidek Co., Ltd., Gamagori, Japan). The imaging and microperimetry examinations had all been undertaken within two weeks following the three month loading phase of anti-VEGF therapy.

Exclusion criteria comprised existing or previous ocular disease other than PCV; significant lenticular opacity; intraocular pressure greater than 21 mmHg; previous ocular trauma or surgery; diabetes; intracerebral disorder; medical therapy known to cause visual field loss; family history of glaucoma; and a spherical refractive error worse than +/−4.00 dioptres and/or cylindrical error worse than +/−3.00 dioptres.

All patients were undergoing de novo anti-VEGF therapy. Twenty-three patients exhibited PCV in one eye, only; the remaining two had bilateral PCV and in these, the treated eye was selected for the study.

### Imaging

ICGA and fluorescein angiography images were centred at the fovea (out to 30° eccentricity; 1536 × 1536 pixels). The SD-OCT images comprised line (100 averaged frames) and volume scans of the central retina (30° × 10° to 30° × 20°; 13 to 31 B-scans; 30–64 averaged frames per B-scan; >22 dB image quality).

**Figure 4:**
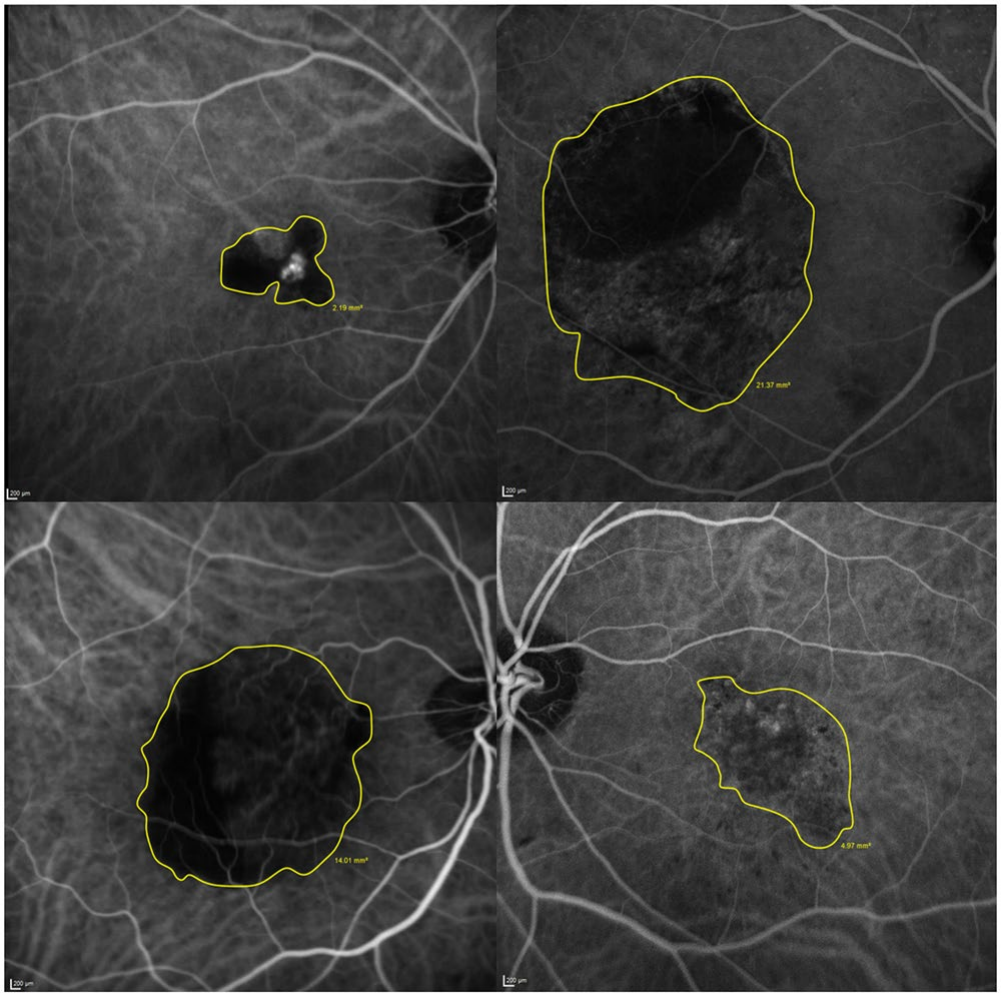
The delineation of the polypoidal choroidal vasculopathy lesion extent by ICGA imaging in four cases.

The polypoidal lesion was delineated from early phase ICGA images using the freehand drawing tool (Heidelberg Eye Explorer software version 1.8.6.0; Heidelberg Engineering, Heidelberg, Germany; Fig. 4). The total lesion area encompassed all the polyps and the abnormal vascular channels^27^. The demarcation of the ICGA images was undertaken by one author and confirmed by a second; any discordance was resolved by a third author.

**Figure 5:**
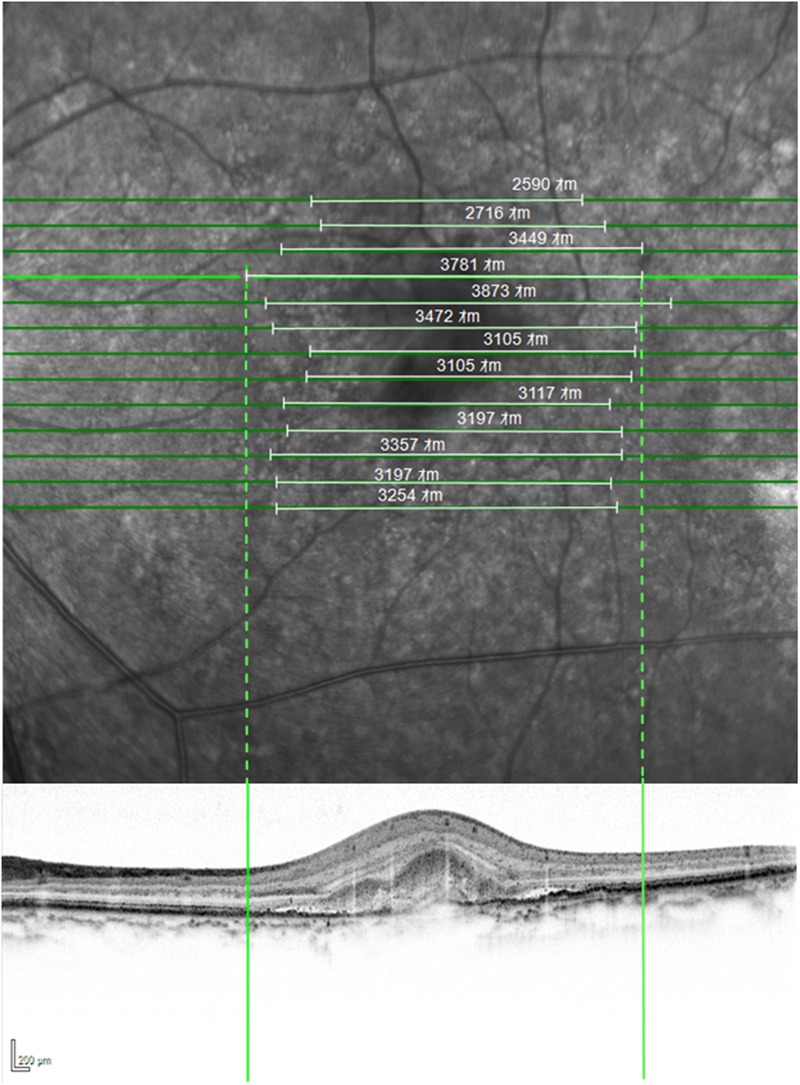
The delineation of the PCV lesion extent by SD-OCT imaging. (Top) The individual volumetric SD-OCT B-scans (horizontal solid green lines) superimposed upon the en-face infrared fundus image of an eye with PCV. The horizontal areal extent of the lesion is demarcated by the solid white lines. (Bottom) An individual SD-OCT B-scan. The areal extent of the lesion is demarcated by the vertical solid green lines and the correspondence to the en-face fundus image by the vertical dotted green lines. SD-OCT = spectral-domain optical coherence tomography; PCV = polypoidal choroidal vasculopathy; m = microns.

The boundary of the PCV lesion was delineated on the SD-OCT images by the use of vertical line markers on each of the B-scans, with simultaneous markings placed on the A-scans (Fig. 5; Heidelberg Eye Explorer software). The boundary encompassed any abnormality consistent with PCV and included abnormal changes of the retinal pigment epithelium. Total retinal thickness measures were obtained out to 7° eccentricity (Iowa Reference Algorithm, Retinal Image Analysis Lab, Iowa Institute for Biomedical Imaging, Iowa City, IA)^28,29^. Subfoveal choroidal thickness was measured from inverted line scans and was available for 21 eyes. The placement of all line markers and the choroidal thickness measures were undertaken by one author and, similarly, confirmed by a second; any discordance was resolved by a third author.

### Microperimetry

Microperimetry was undertaken using a white Goldmann III stimulus, 200 ms stimulus duration, 1.27 cdm^−2^ white background luminance, 127 cdm^−2^ maximum stimulus luminance and a 4-2 dB threshold strategy. The stimulus grid comprised 56 locations extending out to an eccentricity of 10° ^30^. The spatial resolution of the grid was 1.4° out to 4.2° eccentricity and 2.8° between 4.2° and 9.8° eccentricity; each region contained 28 locations^30^ Reliability was determined in terms of the number of incorrect responses to the false-positive catch trials (<15%) (See Supplementary File 2).

The outcome from microperimetry was described in terms of the TD and PD probability levels and is defined in Supplementary File 2. Visual field loss was defined as 3 or more contiguous locations exhibiting PD probability levels of ≤5%^31^.

### Analysis

**Figure 6:**
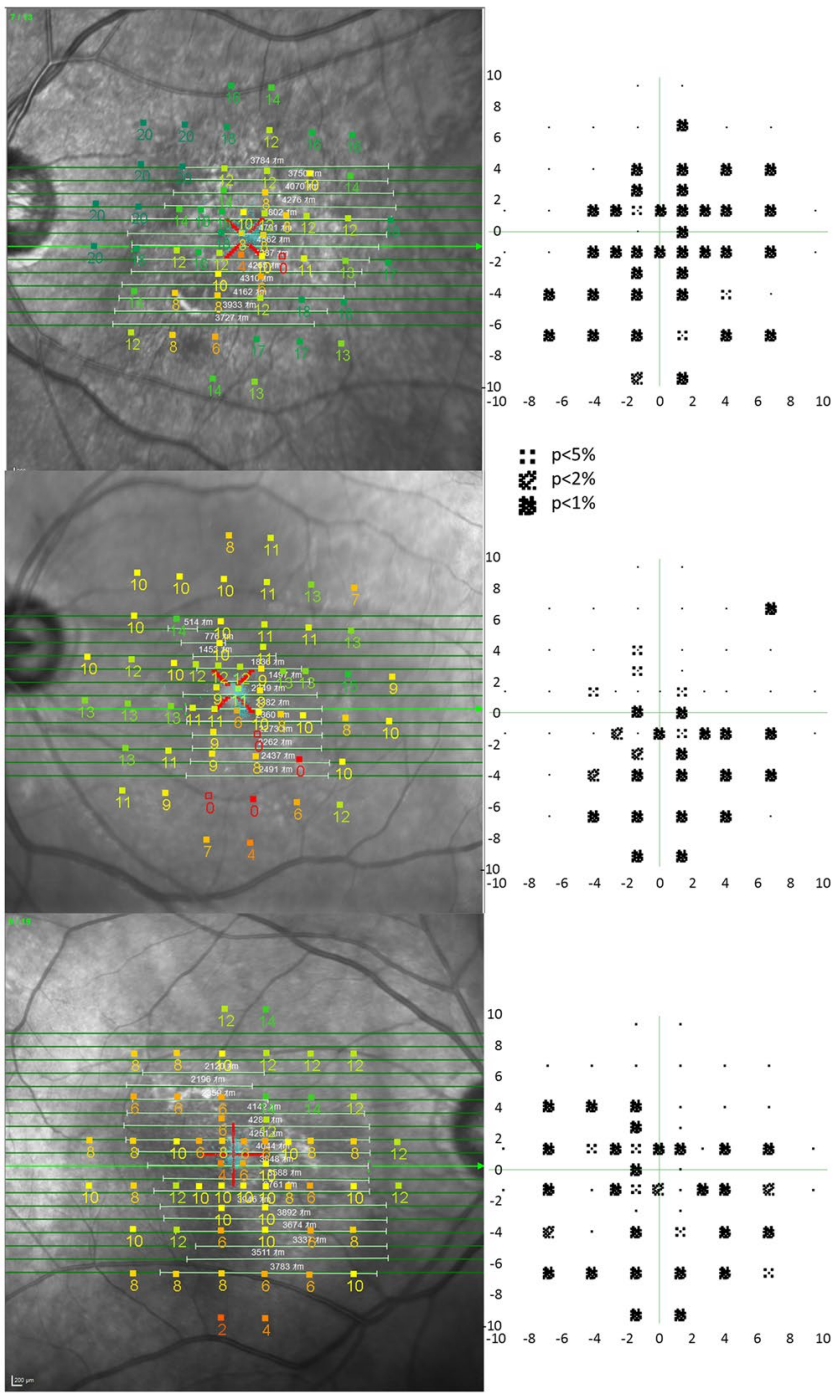
Microperimetry outcomes. (Left) The differential light sensitivity (dB; DLS) at each of 56 stimulus locations is superimposed upon the spectral-domain optical coherence tomography en-face infrared fundus image. The lesion is demarcated (horizontal white lines) from the volumetric B-scans (horizontal green lines). The DLS value is colour coded, using a continuous scale of colour change, to arbitrarily indicate normal (green), borderline (yellow or orange) and abnormal outcomes (red). This colour coding does not take into account the variation of DLS in the normal eye with age or with eccentricity. (Right) The corresponding visual field Pattern Deviation probability maps derived for the study, were referenced to normative data (i.e. accounting for age and eccentricity). Locations exhibiting an abnormal sensitivity at a probability level of <5% < 2% or <1%, respectively, are indicated. The axes show the eccentricity in degrees. m = microns.

The areal extent of the visual field loss was superimposed upon the line-marked en face SD-OCT areal measure using NAVIS MP-1 software (version 1.7.3; Nidek Co., Ltd.) (Fig. 6). The ICGA lesion area was then superimposed onto the combined perimetric and SD-OCT areas.

The areal extents of the PCV lesion by ICGA and by SD-OCT and of the field loss were each expressed in terms of the number of affected locations corresponding to the stimulus grid used for microperimetry. For any given location, the PCV lesion was considered to be present by ICGA and by SD-OCT if the lesion was visible within half the inter-stimulus separation of the stimulus grid, i.e. within a radius of 0.7° at eccentricities of less than 5°, and within a radius of 1.4° between 5° and 10°. At the transition from an inter-stimulus separation of 1.4° to 2.8°, the area of consideration was elliptical. The maximum area was constrained out to 10° horizontally due to the maximum extent of the microperimetry grid and to either 5° or 10° vertically, depending upon the maximum extent of the SD-OCT volume scan.

The concordance in the areal extent of the abnormality between all three modalities was expressed in proportionate terms as the area of mutual overlap relative to the maximum total abnormal area by the three modalities. The concordance between any two modalities was expressed relative to the maximum total abnormal area by both modalities.

The magnitude of any difference in the areal extent of abnormality by any one modality compared to another was expressed as the relative complement of that modality as a proportion of the union of the two modalities under comparison (Supplementary File 1). A greater extent of abnormality was defined, arbitrarily, as >20% of the union of the two given modalities under comparison.

The study was approved by the Kyoto University Graduate School of Medicine Institutional Review Board and all research was performed in accordance with the relevant guidelines. The Board waived obtaining written informed consent in this retrospective study. Information about the study plan was announced on the internet homepage of the Department of Ophthalmology and Visual Sciences at Kyoto University Graduate School of Medicine.

## Electronic supplementary material

Supplementary Files

## Data Availability

The datasets generated during the current study are available from the corresponding author on reasonable request.

## References

[1] Yannuzzi LA, Sorenson J, Spaide RF, Lipson B (1990). Idiopathic polypoidal choroidal vasculopathy (IPCV). Retina.

[2] Spaide RF, Yannuzzi LA, Slakter JS, Sorenson J, Orlach DA (1995). Indocyanine green videoangiography of idiopathic polypoidal choroidal vasculopathy. Retina.

[3] Khan S, Engelbert M, Imamura Y, Freund KB (2012). Polypoidal choroidal vasculopathy: simultaneous indocyanine green angiography and eye-tracked spectral domain optical coherence tomography findings. Retina.

[4] Ciardella AP, Donsoff IM, Huang SJ, Costa DL, Yannuzzi LA (2004). Polypoidal choroidal vasculopathy. Surv Ophthalmol.

[5] Yannuzzi LA (1999). Polypoidal choroidal vasculopathy and neovascularized age-related macular degeneration. Arch Ophthalmol.

[6] Alshahrani ST, Al Shamsi HN, Kahtani ES, Ghazi NG (2014). Spectral-domain optical coherence tomography findings in polypoidal choroidal vasculopathy suggest a type 1 neovascular growth pattern. Clin Ophthalmol.

[7] Coscas G (2015). Toward a specific classification of polypoidal choroidal vasculopathy: idiopathic disease or subtype of age-related macular degeneration. Invest Ophthalmol Vis Sci.

[8] Mori K (2010). Phenotype and genotype characteristics of age-related macular degeneration in a Japanese population. Ophthalmology.

[9] Jirarattanasopa P (2012). Choroidal thickness, vascular hyperpermeability, and complement factor H in age-related macular degeneration and polypoidal choroidal vasculopathy. Invest Ophthalmol Vis Sci.

[10] Coscas G (2014). Comparison of exudative age-related macular degeneration subtypes in Japanese and French Patients: multicenter diagnosis with multimodal imaging. Am J Ophthalmol.

[11] De Salvo G, Vaz-Pereira S, Keane PA, Tufail A, Liew G (2014). Sensitivity and specificity of spectral-domain optical coherence tomography in detecting idiopathic polypoidal choroidal vasculopathy. Am J Ophthalmol.

[12] Cheung CM (2017). Characterization and Differentiation of Polypoidal Choroidal Vasculopathy Using Swept Source Optical Coherence Tomography Angiography. Retina.

[13] Cho HJ (2016). Intravitreal Aflibercept and Ranibizumab Injections for Polypoidal Choroidal Vasculopathy. Am J Ophthalmol.

[14] Oishi A (2015). One-year result of aflibercept treatment on age-related macular degeneration and predictive factors for visual outcome. Am J Ophthalmol.

[15] Koh A (2017). Efficacy and Safety of Ranibizumab With or Without Verteporfin Photodynamic Therapy for Polypoidal Choroidal Vasculopathy: A Randomized Clinical Trial. JAMA ophthalmology.

[16] Imasawa M, Tsumura T, Sekine A, Kikuchi T, Iijima H (2009). Photodynamic therapy for polypoidal choroidal vasculopathy: baseline perimetric results and visual outcomes. Jpn J Ophthalmol.

[17] Ogino K (2013). Intravitreal injection of ranibizumab for recovery of macular function in eyes with subfoveal polypoidal choroidal vasculopathy. Invest Ophthalmol Vis Sci.

[18] Yodoi Y (2007). Central retinal sensitivity measured with the micro perimeter 1 after photodynamic therapy for polypoidal choroidal vasculopathy. Am J Ophthalmol.

[19] Kimura S (2017). Retinal sensitivity after displacement of submacular hemorrhage due to polypoidal choroidal vasculopathy: effectiveness and safety of subretinal tissue plasminogen activator. Jpn J Ophthalmol.

[20] Machida S, Nishimura T, Tamada K, Harada T, Kurosaka D (2012). Macular function evaluated by focal macular electroretinograms after reduced fluence photodynamic therapy in eyes with polypoidal choroidal vasculopathy. Doc Ophthalmol.

[21] Takayama K (2016). Short-term focal macular electroretinogram of eyes treated by aflibercept & photodynamic therapy for polypoidal choroidal vasculopathy. Graefes Arch Clin Exp Ophthalmol.

[22] Wong CW (2016). Age-related macular degeneration and polypoidal choroidal vasculopathy in Asians. Prog Retin Eye Res.

[23] Shin JY, Kwon KY, Byeon SH (2015). Association between choroidal thickness and the response to intravitreal ranibizumab injection in age-related macular degeneration. Acta Ophthalmol.

[24] Chung SE, Kang SW, Lee JH, Kim YT (2011). Choroidal thickness in polypoidal choroidal vasculopathy and exudative age-related macular degeneration. Ophthalmology.

[25] Johnson CA, Sample PA, Cioffi GA, Liebmann JR, Weinreb RN (2002). Structure and function evaluation (SAFE): I. criteria for glaucomatous visual field loss using standard automated perimetry (SAP) and short wavelength automated perimetry (SWAP). Am J Ophthalmol.

[26] Wu Z, Ayton LN, Guymer RH, Luu CD (2013). Intrasession test-retest variability of microperimetry in age-related macular degeneration. Invest Ophthalmol Vis Sci.

[27] Tan CS (2015). EVEREST study report 2: imaging and grading protocol, and baseline characteristics of a randomised controlled trial of polypoidal choroidal vasculopathy. Br J Ophthalmol.

[28] Abramoff MD, Garvin MK, Sonka M (2010). Retinal imaging and image analysis. IEEE reviews in biomedical engineering.

[29] Kang L, Wu X, Chen DZ, Sonka M (2006). Optimal surface segmentation in volumetric images–a graph-theoretic approach. IEEE transactions on pattern analysis and machine intelligence.

[30] Ogino K (2011). Evaluation of macular function using focal macular electroretinography in eyes with macular edema associated with branch retinal vein occlusion. Invest Ophthalmol Vis Sci.

[31] Hudson C (1998). Short-wavelength sensitive visual field loss in patients with clinically significant diabetic macular oedema. Diabetologia.

