# Supplementary material for: Microperimetry and multimodal imaging in polypoidal choroidal vasculopathy
Source: Sci Rep. 2018 Oct 25;8:15769. doi: 10.1038/s41598-018-33781-5 (PMC6202363; doi:10.1038/s41598-018-33781-5)
Supplement: Supplementary file 1 — Supplementary Files [file 41598_2018_33781_MOESM1_ESM.pdf]

Supplementary Files

Title: Microperimetry and multimodal imaging in polypoidal choroidal vasculopathy

Authors: Jennifer H. Acton, Ken Ogino, Yumiko Akagi, John M. Wild, Nagahisa Yoshimura

Supplementary File 1.

Schematic representation of the calculation of the areal extent of the given abnormality.

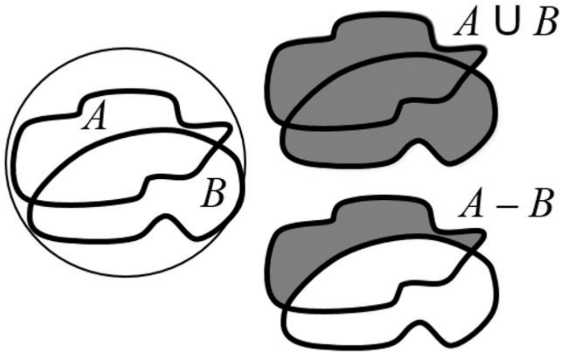

Wider loss (lesion extent) by modality A is defined as:

$$\left\{\left(\frac{A - B}{A \cup B}\right) \times 100\right\} > 10$$

Supplementary File 2.

Microperimetry: definition of visual field loss

The outcome from microperimetry at each stimulus location for each individual was expressed in terms of Pattern Deviation (PD) analyses. Firstly, the Total Deviation (TD) was derived by subtracting the age-corrected normal value from the measured differential light sensitivity.[1] The distributions of age-corrected normal values were derived by weighted linear interpolation[2] from those corresponding to the Program 10-2 stimulus pattern of the MP-1 microperimeter.[3] Secondly, the PD values were then derived from the TD values by the general height adjustment.[1,4] The resulting PD value at each location was then compared to its associated probability levels. Visual field loss was defined as either a single location exhibiting a probability value of $p \leq 0.01$ by PD probability analysis or at least two contiguous locations each exhibiting a probability value of $p \leq 0.05$. In advanced loss (MD worse than -15dB), the PD values defaulted to the TD values as, in such cases, the former cannot be calculated with any certainty.[4] Four individuals exhibited advanced visual field loss, such that the PD values defaulted to the TD values. The foveal location was excluded from the analysis on the basis of the large response variability at this location with the MP-1 microperimeter.

1 Heijl, A., Lindgren, G. & Olsson, J. A Package for the Statistical Analysis of Visual Fields. *Doc Ophthalmol Proc Ser* **49**, 153-168 (1987).

2 Weber, J. & Geiger, R. in *Perimetry Update 1988/89, Proceedings of the VIIIth International Perimetric Society Meeting, Vancouver, Canada* (ed A. Heijl) 447-454 (Kugler & Ghedini Publ 1989).

3 Acton, J. H., Bartlett, N. S. & Greenstein, V. C. Comparing the Nidek MP-1 and Humphrey Field Analyzer in normal subjects. *Optom. Vis. Sci.* **88**, 1288-1297 (2011).

4 Asman, P., Wild, J. M. & Heijl, A. Appearance of the pattern deviation map as a function of change in area of localized field loss. *Invest Ophthalmol Vis Sci* **45**, 3099-3106, doi:10.1167/iovs.03-0617 (2004).
